# A systems biology-driven approach to construct a comprehensive protein interaction network of influenza A virus with its host

**DOI:** 10.1186/s12879-020-05214-0

**Published:** 2020-07-06

**Authors:** Qurat ul Ain Farooq, Zeeshan Shaukat, Sara Aiman, Tong Zhou, Chunhua Li

**Affiliations:** 1 Faculty of Environmental and Life Sciences, Beijing University of Technology, Beijing, 100124 China; 2 Faculty of Information Technology, Beijing University of Technology, Beijing, 100124 China

**Keywords:** Influenza a virus, Hepatitis C virus, Protein-protein interaction networks, Cytoscape, KEGG

## Abstract

**Background:**

Influenza A virus (IAV) infection is a serious public health problem not only in South East Asia but also in European and African countries. Scientists are using network biology to dig deep into the essential host factors responsible for regulation of virus infections. Researchers can explore the virus invasion into the host cells by studying the virus-host relationship based on their protein-protein interaction network.

**Methods:**

In this study, we present a comprehensive IAV-host protein-protein interaction network that is obtained based on the literature-curated protein interaction datasets and some important interaction databases. The network is constructed in Cytoscape and analyzed with its plugins including CytoHubba, CytoCluster, MCODE, ClusterViz and ClusterOne. In addition, Gene Ontology and KEGG enrichment analyses are performed on the highly IAV-associated human proteins. We also compare the current results with those from our previous study on Hepatitis C Virus (HCV)-host protein-protein interaction network in order to find out valuable information.

**Results:**

We found out 1027 interactions among 829 proteins of which 14 are viral proteins and 815 belong to human proteins. The viral protein NS1 has the highest number of associations with human proteins followed by NP, PB2 and so on. Among human proteins, LNX2, MEOX2, TFCP2, PRKRA and DVL2 have the most interactions with viral proteins. Based on KEGG pathway enrichment analysis of the highly IAV-associated human proteins, we found out that they are enriched in the KEGG pathway of basal cell carcinoma. Similarly, the result of KEGG analysis of the common host factors involved in IAV and HCV infections shows that these factors are enriched in the infection pathways of Hepatitis B Virus (HBV), Viral Carcinoma, measles and certain other viruses.

**Conclusion:**

It is concluded that the list of proteins we identified might be used as potential drug targets for the drug design against the infectious diseases caused by Influenza A Virus and other viruses.

## Introduction

The first case of human infection with Influenza A Virus (IAV) was reported in Hong Kong in 1997. Since then, the virus has spread from South East Asia to Europe and Africa, and now it has become a serious threat to human life causing 250,000–500,000 deaths annually worldwide [1–3]. Although the risk of IAV virus to humans is sporadic, it has the potential to produce a severe pandemic because of its plethora of strains and strong capability to transmit easily from person to person [4–6]. Influenza A virus is a negative-sense RNA virus which belongs to the family *Orthomyxoviridae* [7]. Its genome comprises eight segments, each of which encodes one or more proteins. The ten conventional proteins are M1, M2, PB1, PB2, NS1, NS2, NA, HA, PA and NP. In addition to these, IAV virus encodes several other proteins with complementary and frameshift sequences [4, 7, 8].

Protein interaction networks can help researchers gain innumerable insights into functional organizations of proteomes, life cycles of pathogens and molecular pathogen-host relationship. As a virus relies heavily on the host cellular machinery for its replication, the established intermolecular interactions between virus and host are probably necessary for its propagation [9]. Thus based on the study of virus-host protein-protein interactions, scientists can find out the essential host factors that are enriched in the infectious pathway and might be used as potential drug targets [10, 11]. In our previous study on the protein interaction network of Hepatitis C virus (HCV) with host *Homo sapiens*, we did find out some important proteins that might serve as potential drug targets for the treatment of HCV infections.

The goal of our study is to construct a comprehensive protein interaction network of IAV with human host by integrating both small-scale and large-scale researches carried out experimentally or computationally. Based on the network, the IAV-host relationship can be explored at a molecular level in terms of the biochemical pathways, and the important human proteins can be dug out which are highly involved in the IAV infectious pathway.

## Materials and methods

### Data collection

The first step of this study is to get a state-of-the-art picture of the studies on IAV specifically in the context of protein-protein interactions. In order to obtain the related literature, an advanced search for articles is performed in PubMed using multiple keywords restricted to protein-protein interactions between IAV and *Homo sapiens*. As a result, we got 2005 research papers involving IAV-human protein-protein interactions, and among them 19 papers contain the protein-protein interaction data, as shown in Table 1. Besides the data, we also collected the protein-protein interaction data from databases including VirusMINT, IntAct and VirusMentha where the data were obtained using a variety of high-throughput protein-protein interaction detection methods including yeast two-hybrid, co-immunoprecipitation and mass spectrometry, and some computational methods.

**Table 1:** List of the papers and the numbers of IAV-human protein-protein interactions reported in them

No.	Paper	No. of Interactions	References
1.	Pereira, 2018	3	[12]
2.	García, 2018	1093	[13]
3.	Kordyukova, 2018	4	[14]
4.	Kamal, 2017	5	[8]
5.	Mok, 2017	3	[15]
6.	Zhao, 2017	136	[6]
7.	Wang, 2017	1	[16]
8.	Kuo, 2016	64	[17]
9.	Sun, 2015	132	[18]
10.	Cheong, 2015	1	[19]
11.	Gao, 2015	1	[20]
12.	York, 2014	2	[21]
13.	Engel, 2013	8	[22]
14.	Tripathi, 2013	1	[23]
15.	Fournier, 2012	8	[24]
16.	Guan, 2012	8	[25]
17.	Mok, 2012	1	[26]
18.	Demirov, 2012	1	[27]
19.	Yan, 2010	5	[28]

From the above resource, we selected the IAV-human protein-protein interactions that were detected using either small-scale or large-scale experimental methods. In order to get the maximum interaction data, every single association between proteins from IAV and human respectively was gathered from the experimental results. The data are in different formats as researchers use different molecular identifiers (such as Uniprot, EMBL/GenBank and database-specific IDs) in their studies.

### Network construction and analysis

Cytoscape [29] (version 3.7.1) is used to construct and visualize the IAV-human protein-protein interaction network. Cytoscape is a popular, user-friendly and freely available platform for the construction and exploration of biomolecular networks, and it has over 100 built-in tools including Network Analyzer [30], MCODE [31], CytoHubba [32] and CytoCluster [33]. With the Network Analyzer tool, the topological parameters of a network can be calculated which include the network diameter, the average number of neighbors, clustering coefficient, characteristic path length and so on. CytoHubba is an important plugin for the analysis of biomolecular interactions involved in the process of cellular metabolism or the process of signal transduction. With CytoHubba, one can compute different scores for protein nodes like degree, Maximum Neighborhood Component, Edge Percolated Component and Maximal Clique Centrality, sort the nodes based on these scores, and then make subgraphs of top-ranked nodes. It should be noted that all the data used to construct a network in Cytoscape need to be in one clean format. Here, Uniprot ID mapping tool [34] (www.uniprot.org/mapping/) is used to convert the interaction data into the same gene name format.

### Node clustering within the network

Cytoscape has various plugins for clustering nodes including MCODE, SCODE [35], ClusterOne [36], ClusterViz [37], CytoCluster [33] and ClusterMaker [38] which are based on different clustering algorithms. These plugins have shown to be effective in finding important protein complexes and densely connected nodes. The aim of the plugins is to detect valuable functional motifs and then isolate them as sub-networks from the overall protein interaction network. In this study, MCODE, CytoCluster, ClusterOne and ClusterViz are used to identify the protein clusters within the IAV-human protein interaction network.

### GO and KEGG pathway analyses

Kyoto Encyclopedia of Genes and Genomics (KEGG) [39] -based pathway analysis is performed for the top highly IAV-interacting host proteins (hubs) to find out the potential pathways where the group of genes are enriched. The tool used for this purpose is Enrichr [40], an open-source integrative and web-based resource for enrichment analysis, which contains over 70,000 annotated gene sets organized into over 100 gene-set libraries. In addition to KEGG, Gene Ontology (GO) [41] -based analysis is also performed through which the biological processes, cellular components and molecular functions can be identified where the set of crucial genes are involved.

In this research, we incorporate the current work and our previous study on the protein interaction network of HCV with *Homo sapiens*. By comparing the results from the two studies, we want to identify the potential common host factors involved in both of the disease pathways. The KEGG-based pathway analysis is also performed for the common host proteins in order to find out the potential pathways in which those gene products are actively enriched.

## Results

### Protein interaction network of IAV with *Homo sapiens*

**Fig. 1:**
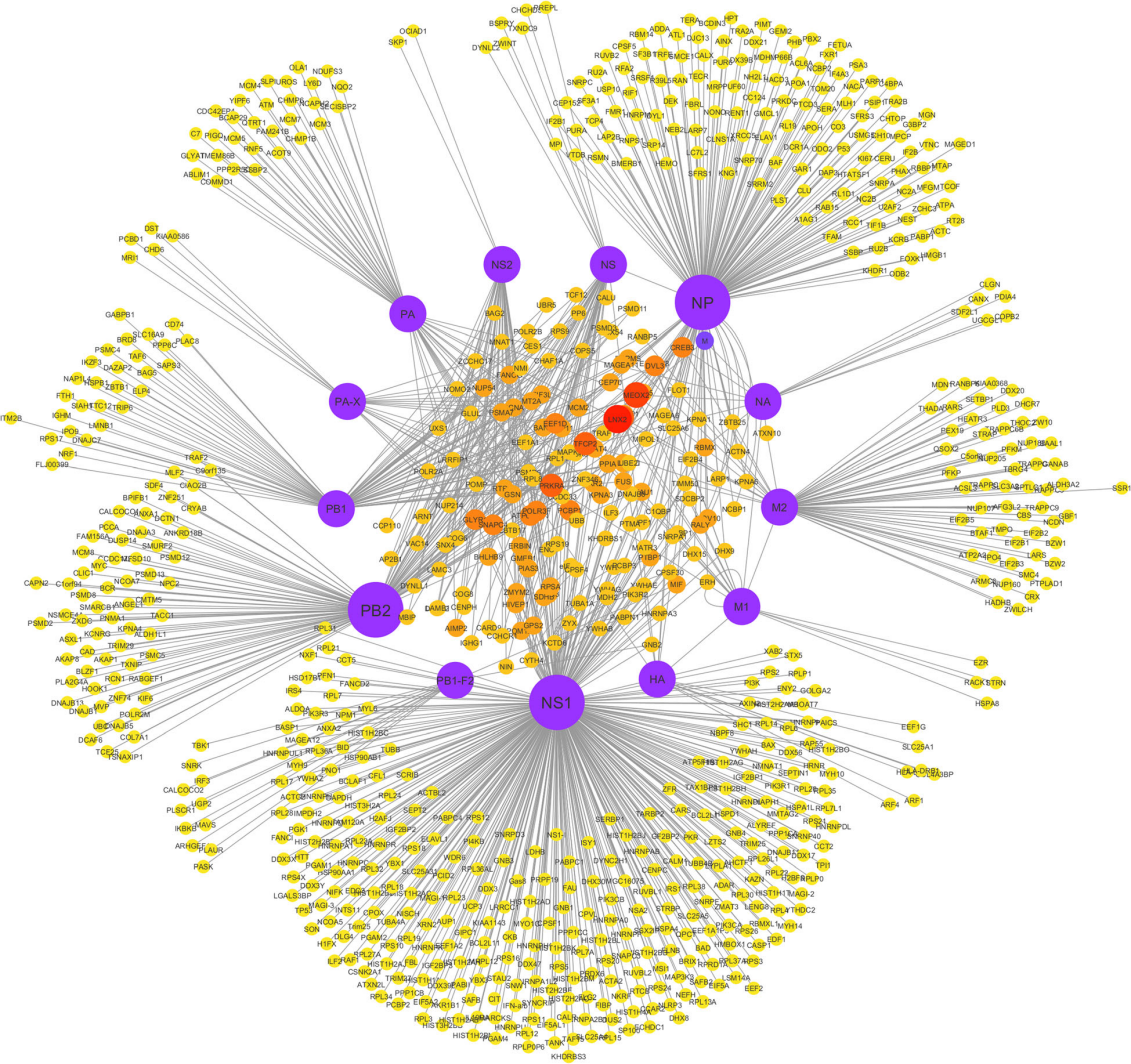
Protein-protein interaction network of Influenza A virus with host *Homo sapiens* constructed in Cytoscape. The network contains 829 nodes (proteins) among which 14 are viral proteins while 815 are human proteins. Total number of edges (interactions) are 1051 and the highly connected nodes tend to make clusters and hubs in the network. Viral proteins NS1, NP and PB2 are shown in a bigger size due to their higher numbers of associations with human proteins. The average number of interactions for a node is 2.4, which means the network is not very dense

**Fig. 2:**
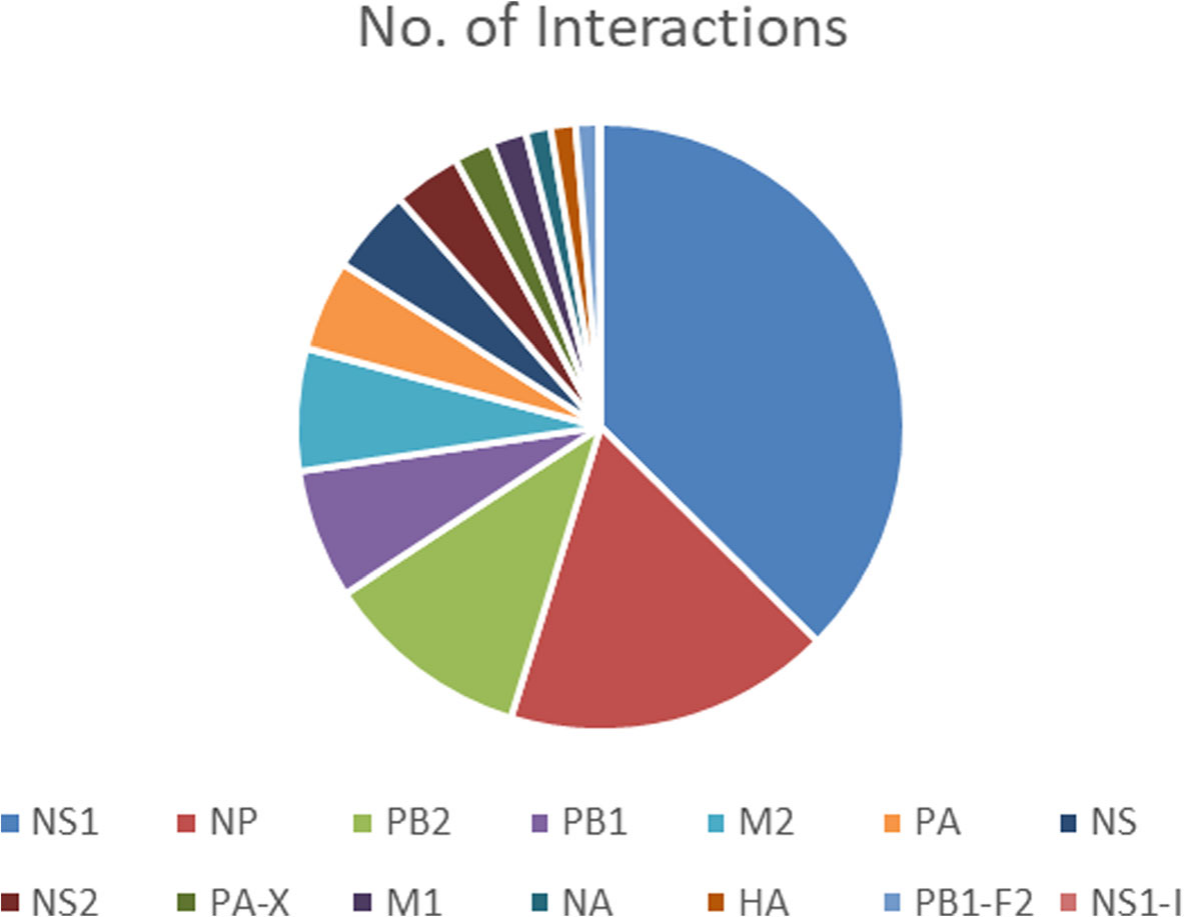
A pie chart of IAV proteins based on their numbers of interactions with human proteins

**Fig. 3:**
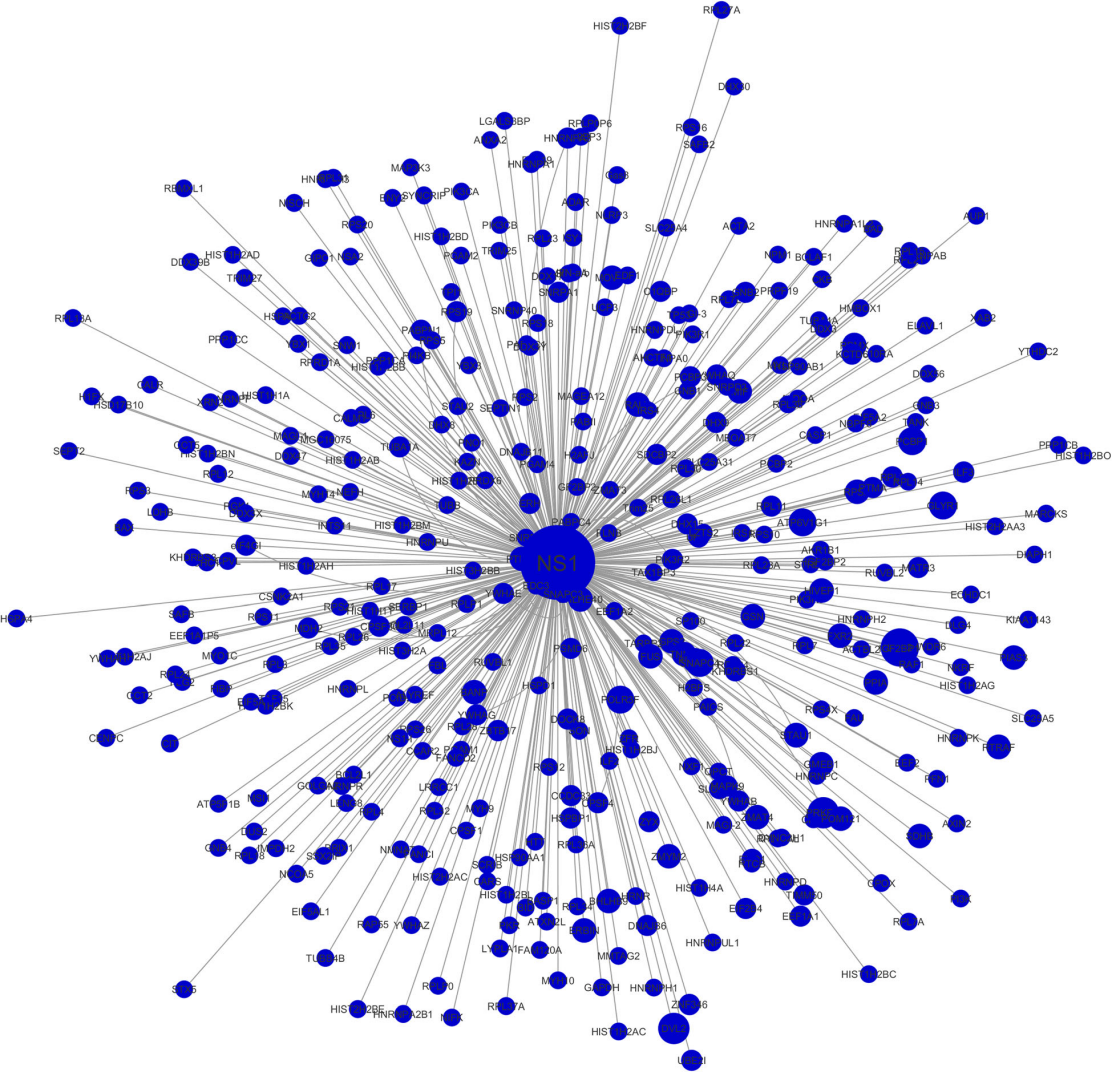
A highly connected subgraph formed between viral protein NS1 with human proteins

After integrating all the interaction data from literature and databases, the total number of protein interactions between IAV and *Homo sapiens* is 1477. After removal of the duplicate interactions, this number becomes 1027. Figure 1 shows the protein-protein interaction network constructed in Cytoscape. The number of nodes in the network is 829 among which 14 are viral proteins while 815 belong to human proteins. The viral protein NS1 has the highest number of interactions (397) with human proteins, followed by NP and PB2 with 184 and 117 associations respectively. Based on the network, we utilized CytoHubba to rank individual nodes by different features as described in the section of Materials and Methods. Figure 2 represents the rankings of viral proteins based on their respective numbers of associations with host proteins (see Table 2 for detailed data). Furtherly, CytoCluster [33] was used to cluster the nodes, and from the result we found out the highly connected hubs and potential protein complexes in the biological networks. Figure 3 represents a single highly connected cluster produced from ClusterOne algorithm, one of the six algorithms in CytoCluster. Through network analyzer tools, we also obtained important statistical features of the network. Table 3 displays some important statistical parameters of the protein-protein interaction network between IAV and human.

**Table 2:** List of the IAV proteins with the most interactions with human proteins

IAV Proteins	No. of Interactions	Percentage
NS1	397	37%
NP	184	17%
PB2	117	11%

**Table 3:** Important statistical parameters of the network

Attributes	Values
No. of Nodes	829
No. of Edges	1051
Avg. number of neighbors	2.475
High interacting proteins in IAV	NS1, NP, PB2
Network Density	0.003
Network Diameter	6
Clustering Coefficient	0.001
Shortest Paths	673,252 (98%)

### KEGG and GO enrichment analyses of top-notch IAV-associated host proteins

**Fig. 4:**
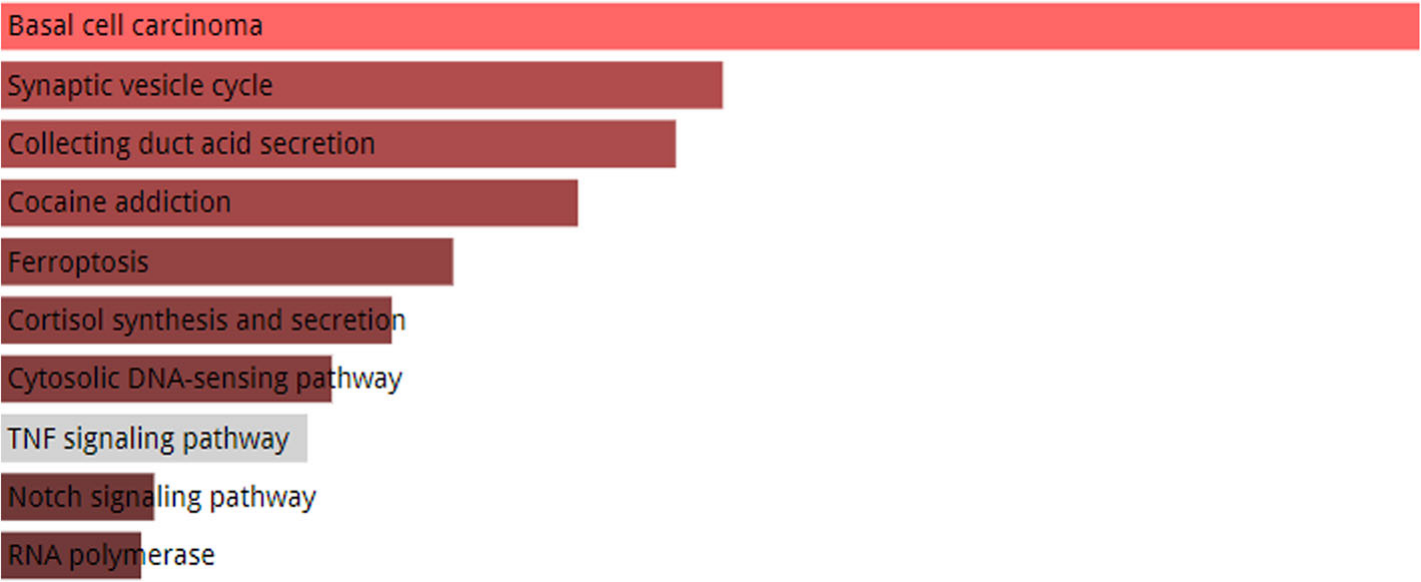
KEGG pathways in which the 13 highly IAV-interacting host factors are enriched. The color of the bar shows the intensity of the gene set enriched in a specific pathway. The lighter the color, the more enriched the genes are in that pathway

Among the host proteins that have interactions with IAV proteins, we found that certain human proteins are associated with more viral proteins compared with the others. Human proteins LNX2 and MEOX2 interact with 7 and 6 viral proteins respectively. Host proteins TFCP2, PRKRA and DVL2 were found to be interacting with 5 viral proteins respectively while eight proteins are associated with 4 viral proteins each. Figure 4 shows the resulting graph of KEGG pathway analysis for the top 13 IAV-interacting host proteins. From Fig. 4, it can be seen that the set of genes are highly enriched in the pathway of basal cell carcinoma which is a type of skin cancer. The other pathways in which the gene set is enriched are the pathways of synaptic vesicle cycle, collecting duct acid secretion, cocaine addiction, ferroptosis, and cortisol synthesis and secretion. Gene Ontology analysis for these highly IAV-interacting host proteins shows that the gene products are involved in the important biological processes including snRNA transcription from RNA polymerase III promoter, beta-catenin destruction complex disassembly, response to increased oxygen levels, production of siRNA involved in RNA interference, cellular response to oxygen levels, and certain other processes. The complete depiction of the Gene Ontology information i.e. biological processes, molecular functions and cellular components is shown in Figures S1, S2, S3 in supplementary materials.

### Node clustering analysis

**Fig. 5:**
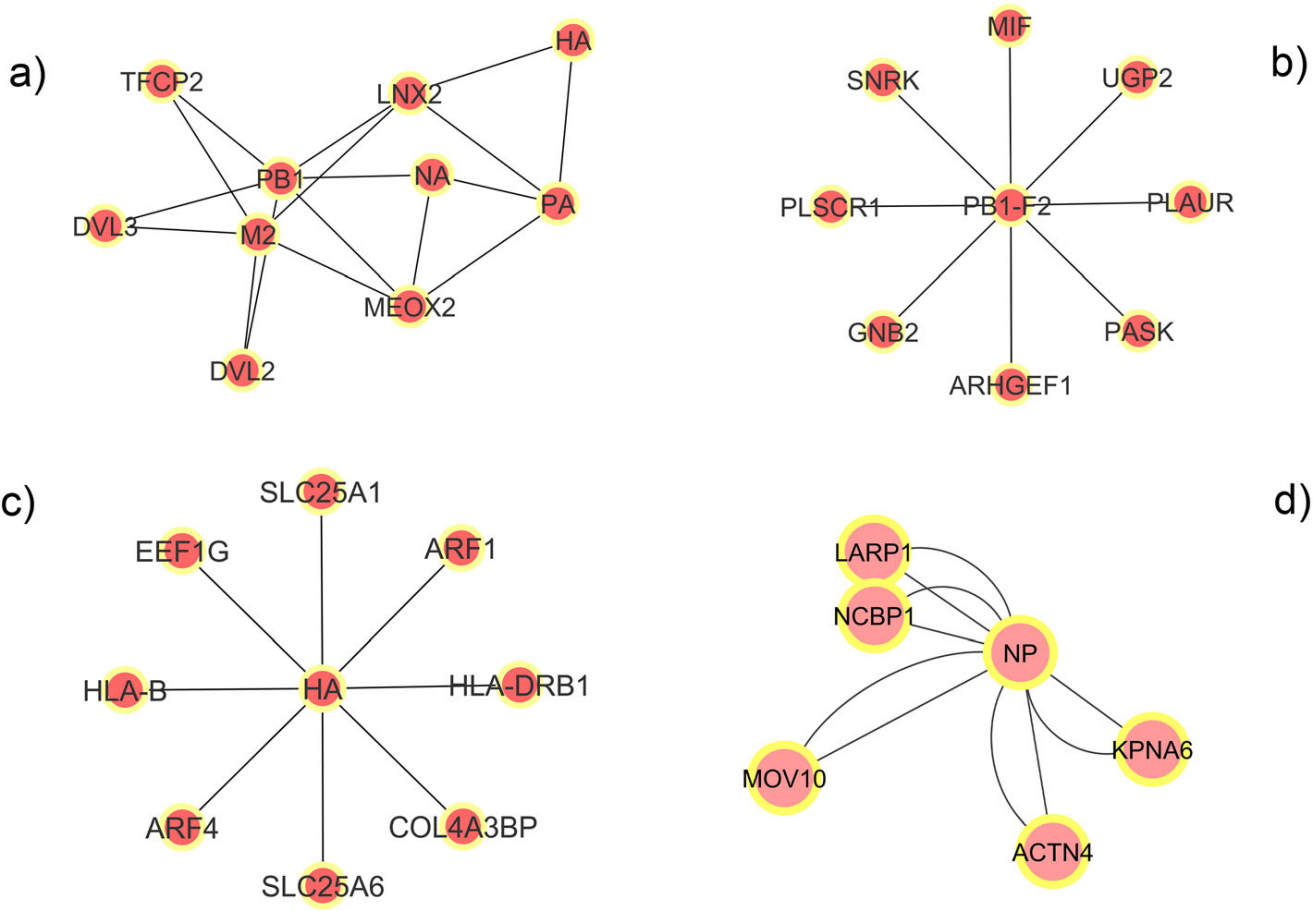
Node clusters made for IAV-human protein interaction network in Cytoscape with different clustering algorithms including MCODE a), CytoCluster b), ClusterViz c) and ClusterOne d)

In a protein interaction network, node clusters, highly interconnected node groups, generally consist of two classes of modules i.e. protein complexes and functional modules. With node clustering analysis, researchers have identified some significant functional modules in the protein-protein interaction studies on Type 2 Diabetes [42] and Burkitt’s lymphoma [43] respectively. In order to detect the potential protein complexes and functionally important modules in IAV-human protein interaction network, node clustering was performed with different methods including the hierarchical, k-means, k-medoid, density-based and spectral-based clustering algorithms. The hierarchical clustering algorithm builds a tree that connects nodes in the network hierarchically while the density-based algorithm detects densely connected subgraphs in the network. Figure 5 shows multiple clusters made for the IAV-human protein interaction network by MCODE, CytoCluster, ClusterViz and ClusterOne respectively. The cluster detected by MCODE has 10 nodes in which HA, NA, PA, PB1 and M2 belong to viral proteins while LNX2, TFCP2, DVL2, DVL3 and MEOX2 belong to human. In the cluster made by CytoCluster, there are 9 nodes among which PB1-F2 belongs to viral proteins while MIF, UGP2, PLAUR, PASK, ARHGEF1, GNB2, PLSCR1 and SNRK are human proteins. There are 9 nodes in the cluster detected by ClusterViz where HA is a viral protein while SLC25A1, ARF1, HLA-DRB1, COL413BP, SLC25A6, ARF4, HLA-B and EEF1G belong to human. The cluster made by ClusterOne has 6 nodes with NP as a viral protein and KPNA6, ACTN4, MOV10, NCBP1 and LARP1 belonging to human. These nodes are densely interconnected to each other but sparsely associated to the other nodes, and the clusters formed by them contain potential protein complexes and functionally important modules in IAV-human protein interaction network.

### Common host factors involved in IAV and HCV infection pathways

**Fig. 6:**
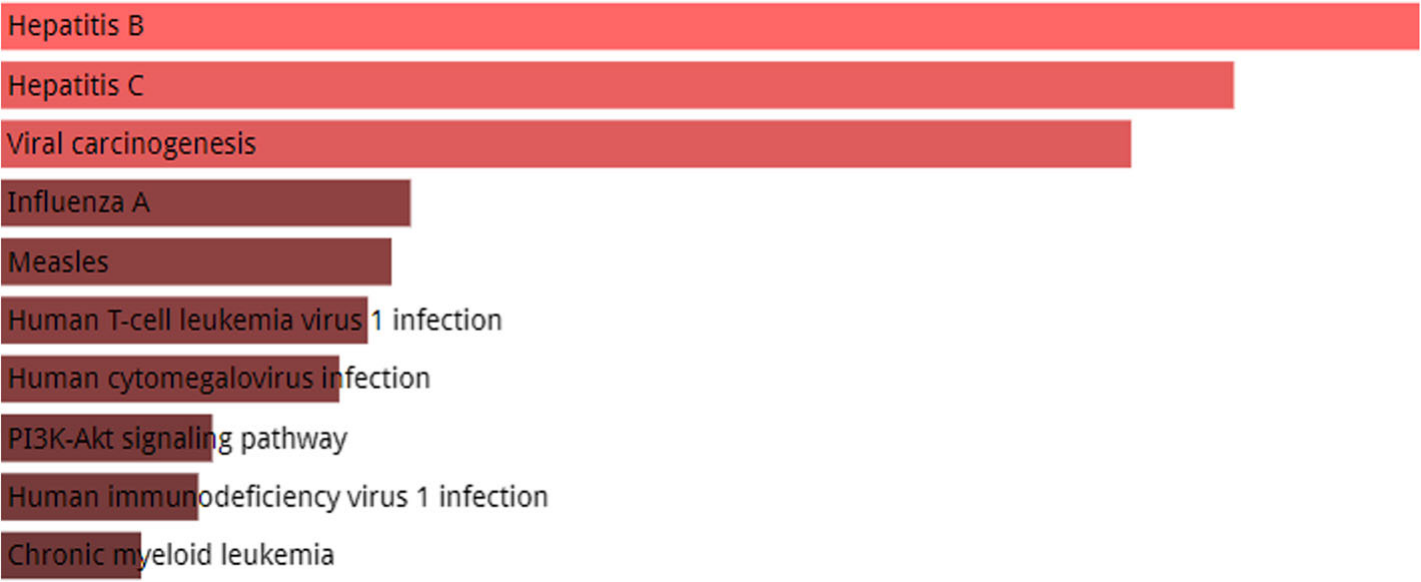
KEGG pathways in which the common host genes are enriched involved in both infectious pathways of IAV and HCV viruses

In our previous study on the protein interaction network of Hepatitis C Virus with *Homo sapiens*, we found out some potential human proteins that interact with HCV viral proteins [44]. Here, by comparing the results from the current and previous studies, we detected common host factors involved in both viral infection pathways. Out of 815 host factors from the current study and 940 identified in the previous study, 138 host proteins were found to overlap in both studies, which means that these gene products are involved in both HCV and IAV infectious pathways. Table S1 gives the list of the 138 common host factors in supplementary materials. Figure 6 shows the results of KEGG pathway analysis for these proteins. It can be seen that these proteins are not only involved in HCV and IAV infectious pathways but also actively enriched in the infectious pathways of Hepatitis B Virus, viral carcinogens, measles, human T-cell leukemia virus 1 and human cytomegalovirus.

## Discussion

Regarding the Influenza A virus, a plethora of strains has been reported to have contrasting transmissibility and pathogenesis. In 2013, an epidemic of a novel reassortant influenza A virus came out in China which caused a life-threatening human infection [4, 45]. To prevent and control influenza, scientists have developed vaccine and immunization strategies to fight against the disease. However, as the strategies need to be updated persistently, they become an expensive, clumsy and complicated process. Moreover, due to the virus’s ability to rapidly mutate and the stubborn nature of new viral strains, the currently available antiviral treatments are losing their efficacy. Although vaccines have been the mainstay for influenza prevention until now, they have little or no effect on the persons older than 65 and on the patients having a compromised immune system. Consequently, there is an urgent need for developing new antiviral therapies and drugs.

The currently available antiviral drugs mainly target the two major classes of IAV proteins: M2 channel and neuraminidase [3, 46–48]. Just as we know, viruses utilize host cellular machinery to propagate, which inversely triggers host immune responses to fight against viral infections. Thus, the important IAV-interacting host proteins are also promising for therapeutic purposes. In previous studies, certain host proteins have been identified as potential virus interacting proteins. Our previous study identified KPNA1 and EIF2AK2 as the IAV-interacting proteins [44] which has been reported in literature too [6].

In current study, we present an idea that the host proteins that potentially interact with multiple viral proteins can be taken as the targets for novel antiviral drug design to prevent the entry and replication of the virus. Watanabe et al. [49] in 2015 also proposed the idea that virus-host interactomes can be taken as the basis for the development of antiviral drugs targeting host cellular factors. This research aims to integrate almost all the protein interaction data obtained from different-scale experimental and computational methods to detect highly IAV-associated host factors for helping scientists to advance the development of new drugs. The current study found out 815 host proteins that potentially interact with IAV. Among them, the top 13 highly IAV-interacting proteins are LNX2, MEOX2, TFCP2, PRKRA, DVL2, POLR3F, SNAPC4, GLYR1, ATP6V1G1, PCBP1, EEF1D, DVL3 and CREB3, which are enriched in the pathway of IAV infection. These proteins are not well studied yet, and therefore they might be taken as potential drug targets for the cure of IAV infection.

Additionally, in the current study we used Cytoscape platform, its important plugins and other tools to explore the IAV-human protein interaction network. The functionally important modules and potential protein complexes were identified in the network. Gene Ontology analysis for the top 13 IAV-interacting human proteins revealed the important biological processes in which they are actively enriched, and their molecular functions and cellular components to which they belong. In our previous study [44], we identified 1325 interactions between 12 HCV and 940 human proteins. By comparing the host proteins associated with IAV and HCV viral proteins respectively, we identified the common 138 human proteins that potentially interact with both IAV and HCV viral proteins. KEGG analysis for these proteins revealed that they are involved in a number of infectious pathways, not only in IAV and HCV infections but also in HBV, viral carcinogens, measles, human t-cell leukemia virus type 1 and human cytomegalovirus infections.

It should be noted that the data we integrated in this study came from a vast variety of literature resources and various notable databases, which were detected by large-scale and small-scale experimental methods. Thus, the constructed comprehensive IAV-human protein interaction network and the identified important host factors can provide much valuable information to scientists for developing new therapeutic strategies to fight against the disease. During the past decades, in spite of the great efforts in drug development, the number of clinically validated drug targets is still very small, only 324 [50]. The reason is that developing a new drug from the unique idea and launching of a complete can usually take 12–15 years and the estimated cost is about $1 billion [50, 51]. We believe that through the deep mining of the huge data from virus-human protein interaction networks, the obtained valuable information can significantly help scientists develop specific new drugs, and meanwhile remarkably reduce the time and costs during the process.

## Conclusions

Influenza caused by Influenza A virus is a global public health problem with seasonal and pandemic characteristics. Scientists are using network biology to dig deep into the host factors responsible for regulation of viral infectious pathways. Researchers can find multiple drug targets from the protein-protein interaction network constructed for viral defection, which can help the antiviral drug discovery. Our current study is centered not only on Influenza A virus-human protein interaction network, but also on the comparison of our current result with that we got in our previous study on Hepatitis C Virus-human protein interaction network. Through the study, we found out important disease pathways involved in IAV, HCV, HBV, measles and viral carcinogen infections. The intensities of the genes enriched in these disease pathways might vary from each other but the information on the pathways still provide chances for scientists to knock down the potential host factors to block infectious pathways for curing the disease.

## Supplementary information

**Additional file 1: Table S1.** Common host factors between IAV and HCV infectious pathways. **Figure S1,S2 and S3**. Results of Gene Ontology Analysis of the top 13 IAV-interacting host proteins, which show the biological processes in which the group of genes are taking part, the molecular functions and cellular components to which they belong respectively.

## Data Availability

All the data generated or analyzed during this study are included in this published article [and its supplementary materials files].

## Abbreviations

DVL2: Segment polarity protein dishevelled homolog DVL-2
GO: Gene ontology
HBV: Hepatitis B virus
HCV: Hepatitis C virus
IAV: Influenza A virus
KEGG: Kyoto encyclopedia of genes and genomics
LNX2: Ligand of numb protein X 2
MEOX2: Homeobox protein MOX-2
PPI: Protein-protein interaction.
PRKRA: Interferon-inducible double-stranded RNA-dependent protein kinase activator A
TFCP2: Alpha-globin transcription factor CP2
Y2H: Yeast 2 hybrid
