# Supplementary material for: A systems biology-driven approach to construct a comprehensive protein interaction network of influenza A virus with its host
Source: BMC Infect Dis. 2020 Jul 6;20:480. doi: 10.1186/s12879-020-05214-0 (PMC7339526; doi:10.1186/s12879-020-05214-0)
Supplement: Supplementary file 1 — Additional file 1: Table S1. Common host factors between IAV and HCV infectious pathways. Figure S1,S2 and S3. Results of Gene Ontology Analysis of the top 13 IAV-interacting host proteins, which show the biological processes in which the group of genes are taking part, the molecular functions and cellular components to which they belong respectively. [file 12879_2020_5214_MOESM1_ESM.pdf]

Supplementary Materials

A systems biology-driven approach to construct a comprehensive protein interaction network of Influenza A Virus with its Host

Qurat ul Ain Farooq[1], Zeeshan Shaukat[2], Sara Aiman[1], Tong Zhou[1], Chunhua Li[1]*

[1] Faculty of Environmental and Life Sciences, Beijing University of Technology, Beijing 100124, China.

[2]Faculty of Information Technology, Beijing University of Technology, Beijing 100124, China.

*Correspondence should be addressed to Chunhua Li (E-mail: chunhuali@bjut.edu.cn)

Table S1. Common host factors between IAV and HCV infectious pathways.

CREB3	PPIA	HNRNPA1	NISCH	LRRCC1	DNAJC7
EEF1G	RAN	RPS2	STRBP	RPL26	HSPB1
STAU1	C1QBP	RPS3	RAF1	RPL23	RPS17
HSPA8	PTMA	RPS12	TRIM27	TP53	LMNB1
IPO4	PRKDC	PIK3R1	BAX	HSPA4	NAP1L4
TBRG4	HACD3	YWHAB	GOLGA2	XAB2	CALU
PTPLAD1	APOH	PIK3CB	LZTS2	CSNK2A1	MAVS
ATXN10	APOA1	ADAR	PI4KB	ANXA2	IRF3
RANBP5	CLU	HNRNPK	SSX2IP	FANCI	TBK1
BZW2	SNX4	YWHAE	GAPDH	PSMA7	IKBKB
DDX20	ENO1	RPS24	NPM1	MT2A	CALCOCO2
SAAL1	ARNT	YWHAZ	PFN1	C7	PLSCR1
SLC3A2	NUP214	YBX1	HSP90AA1	ATM	DNAJB1
CANX	GPS2	EEF1A1	HSP90AB1	YIPF6	MVP
MAGED1	GLYR1	TUBB4B	HSPD1	SECISBP2	SMURF2
KPNA1	CCHCR1	HNRNPU	CKB	RNF5	MYC
USHBP1	ZMYM2	ILF2	CFL1	PCNA	BCR
CEP152	LAMC3	ELAVL1	YWHAQ	DST	PLA2G4A
KHDRBS1	DDX3X	HIST2H2BE	CALR	EIF3L	DCAF6
DHX9	DDX3Y	PABPN1	CCT5	FTH1	ASXL1
PTBP1	SLC25A5	HIST3H2BB	GNB2	RPS9	TRAF2
MATR3	TUBB	ATXN2L	TUBA1A	ZBTB1	C1orf94
DDX5	HNRNPC	XRN2	MYH14	SIAH1	DNAJA3

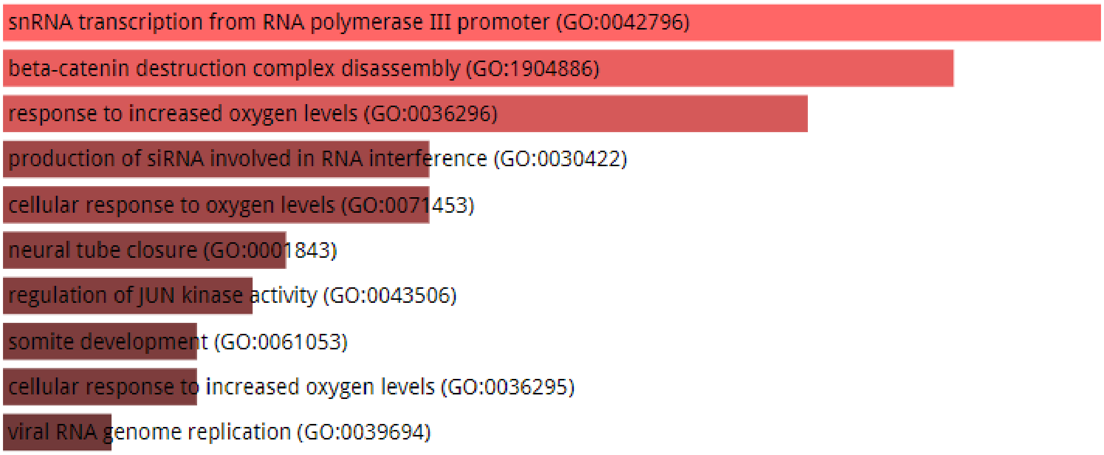

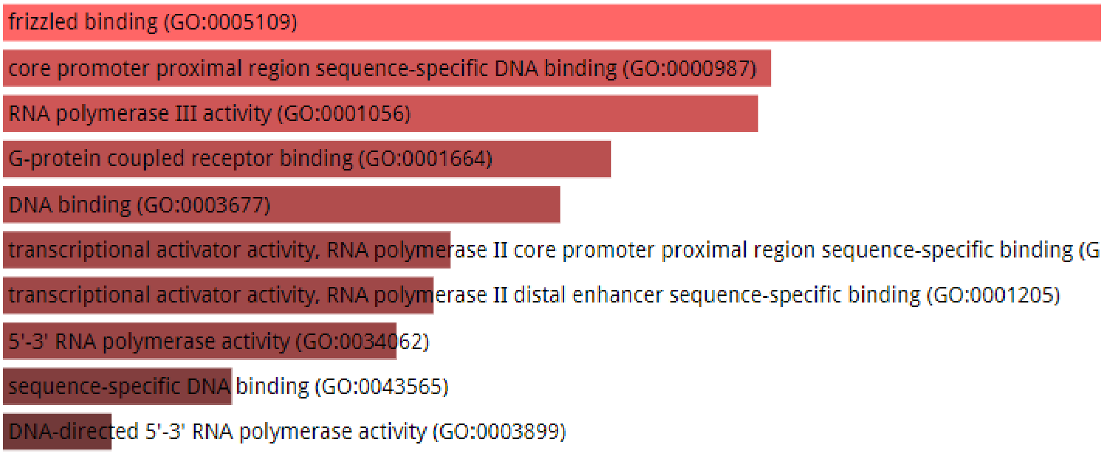

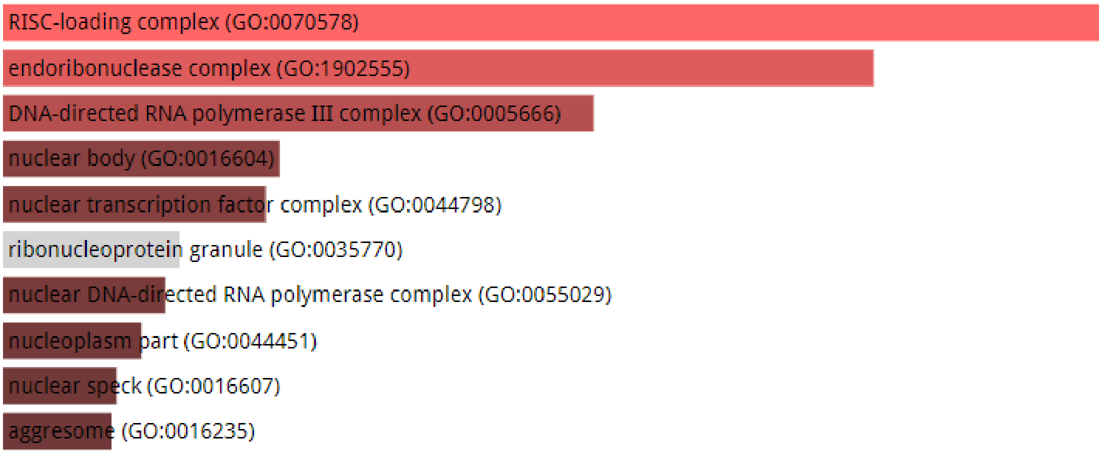

Figure S1,S2 and S3: Results of Gene Ontology Analysis of the top 13 IAV-interacting host proteins, which show the biological processes in which the group of genes are taking part, the molecular functions and cellular components to which they belong respectively.
